# Supplementary material for: Identification of photoperiod-induced specific miRNAs in the adrenal glands of Sunite sheep (Ovis aries)
Source: Front Vet Sci. 2022 Jul 22;9:888207. doi: 10.3389/fvets.2022.888207 (PMC9354845; doi:10.3389/fvets.2022.888207)
Supplement: Supplementary file 1 [file Data_Sheet_1.ZIP › Supplementary file/miRDeep2 file.zip!/miRDeep2 file/Know_Structure/SP42A3/oar-mir-543.pdf]

miRBase precursor : oar-mir-543
Total read count : 7402
oar-miR-543-5p read count : 2
oar-miR-543-3p read count : 7385
remaining reads : 15

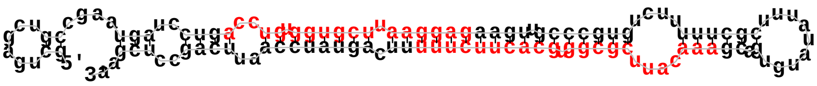

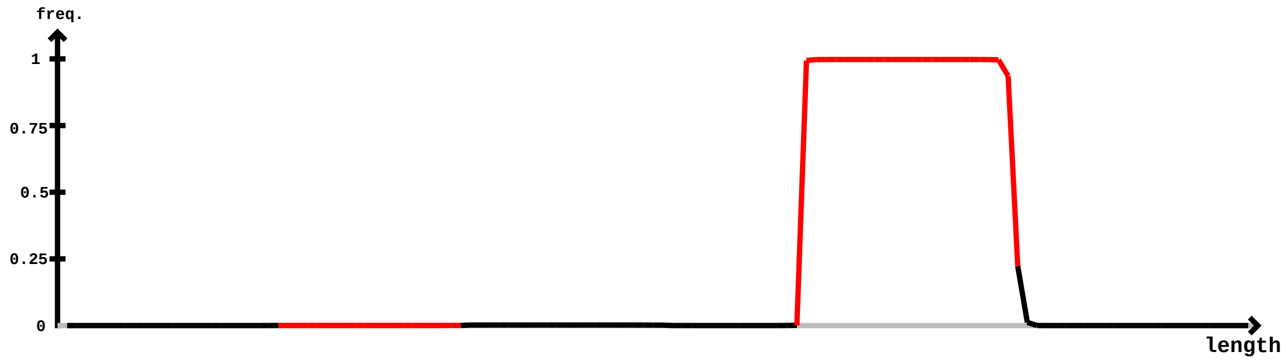

oar-miR-543-3p

oar-miR-543-5p

```
5'- gcugagcugccgaaugauccugaccuguggugcuuaaggagaaguugcccgugucuuuuucgcuuuauaugugacgaaacauucgcggugcacuucuuuuucaguauccaauucagccucgaa  -3'   exp
    ((......)).....((..((((..((.((((((.((((((((((.(((((((....((((((.........).)))))....))))).))))))))))))).))))))))..))))..))...     reads  mm
    ....................ugaccuguggugcuuaagga...................................................................................     1      0
    .....................gaccuguggugcuuaagga...................................................................................     1      0
    .......................................agaaguugcccgugucuuu.................................................................     1      0
    .......................................agaaguugcccgugucuuuuu...............................................................     1      0
    ........................................gaaguugcccgugucuuuuucg.............................................................     1      0
    .........................................aaguugcccgugucuuuuu...............................................................     1      0
    .........................................aaguugcccgugucuuuuucg.............................................................     10     0
    .........................................aaguugcccgugucuuuuucgcuu..........................................................     1      0
    ..........................................................................cgaaacauucgcggugcacuucu..........................     2      0
    ...........................................................................gaaacauucgcggugcacuuc...........................     1      0
    ...........................................................................gaaacauucgcggugcacuucu..........................     3      0
    ...........................................................................gaaacauucgcggugcacuucuu.........................     1      0
    ............................................................................aaacauucgcggugcacu.............................     1      0
    ............................................................................aaacauucgcggugcacuu............................     1      0
    ............................................................................aaacauucgcggugcacuuc...........................     6      0
    ............................................................................aaacauucgcggugcacuucu..........................     443    0
    ............................................................................aaacauucgcggugcacuucuu.........................     5262   0
    ............................................................................aaacauucgcggugcacuucuuu........................     1554   0
    ............................................................................aaacauucgcggugcacuucuuuu.......................     84     0
    ............................................................................aaacauucgcggugcacuucuuuuu......................     2      0
    .............................................................................aacauucgcggugcacuucu..........................     1      0
    .............................................................................aacauucgcggugcacuucuu.........................     14     0
    .............................................................................aacauucgcggugcacuucuuu........................     6      0
    .............................................................................aacauucgcggugcacuucuuuu.......................     2      0
    ..............................................................................acauucgcggugcacuucuu.........................     1      0
    ...............................................................................cauucgcggugcacuucuuu........................     1      0
```
